# Effects of a formalized collaboration between plastic and orthopedic surgeons in severe extremity trauma patients; a retrospective study

**DOI:** 10.1186/s13032-015-0023-4

**Published:** 2015-04-15

**Authors:** Pehr Sommar, Yamin Granberg, Martin Halle, Ann-Charlott Docherty Skogh, Kalle T Lundgren, Karl-Åke Jansson

**Affiliations:** Department of Molecular Medicine and Surgery, Section of Plastic Surgery, Karolinska Institutet and Karolinska University Hospital Stockholm, Stockholm, Sweden; Department of Orthopedic Surgery Västerås Hospital and Department of Molecular Medicine and Surgery, Karolinska Institutet Stockholm, Stockholm, Sweden; Department of Molecular Medicine and Surgery, Section of Orthopedics and Sports Medicine, Karolinska Institutet and Karolinska University Hospital Stockholm, Stockholm, Sweden

**Keywords:** Extremity trauma, Flaps, Orthoplastic collaboration

## Abstract

**Background:**

Severe trauma to the extremities often includes a combination of fractures and soft tissue injuries. Several publications support that the patient outcome is better when skeletal stabilization is followed by early soft-tissue coverage. In an effort to optimize the treatment of these patients, we established a formalized collaboration in 2008 between the Departments of reconstructive plastic surgery and orthopedics at the Karolinska University Hospital.

**Methods:**

A retrospective review was conducted for all patients who had suffered severe extremity trauma and received either a free or a pedicled flap for extremity reconstruction. We compared the management of patients 0–4 years before and 0–4 years after the collaboration started especially with respect to; choice of flap, time to flap coverage, number of operations/revisions, total in-hospital stay.

**Results:**

After initiation of the collaboration, the number of flaps increased from 13 flaps (5 free and 8 pedicled) to 44 flaps (21 free and 23 pedicled). Fewer postoperative revisions was seen, as well as shorter in-hospital stay.

**Conclusions:**

The present study highlights the importance of formalized collaboration between orthopedic and plastic surgeons in severe extremity trauma patients. The concept of an interdisciplinary approach has led to an increased number of trauma patients referred for plastic surgical consultation, an increased number of flaps, fewer postoperative revisions and shorter hospital stay.

## Introduction

Severe extremity trauma is often associated with a combination of fractures and soft tissue injuries. It is generally accepted that the outcome is better when skeletal stabilization is followed by early soft-tissue coverage [1-4]. Soft tissue flaps provide the protection and vascularization needed to resist infection and promote bone healing. Meling et al. reported the incidence of all open long bone fractures to be 13/10^5^/year 2004–2007 [5]. Weiss et al. found a decreasing incidence of open tibial shaft fractures in the Swedish population, which was 2.3/10^5^/year, 1998–2004 [6]. Because of the complexity of these fractures and the low incidence rate, an orthoplastic approach has been suggested; i.e. a multidisciplinary collaboration involving both orthopedic and plastic surgery teams [7,8].

Naique et al. demonstrated that severe open tibia fractures treated at dedicated trauma units with both orthopedic and plastic surgery services had lower complication rates and less need for revision surgery compared with those treated initially at hospitals without such combined services [8].

To ensure an orthoplastic approach, we formalized a collaboration between the Departments of reconstructive plastic surgery and orthopedics in April 2008 (Multidisciplinary collaboration MDC). The collaborative protocol agreed upon early attendance of plastic surgeons in extremity trauma cases, preferably at the trauma room or at primary revisions of open fractures in order to expedite conjoint planning of further operations. An outpatient clinic with orthopedics and plastic surgeons for patients admitted from other hospitals was set up as well as multidisciplinary postoperative follow up of patients with trauma, and osteomyelitis following trauma to the extremities. A contract was established to ensure equal contribution of respective operating facilities and to ensure efficient rescheduling of elective surgery when emergent full day cases were to be planned with short notice.

To investigate the effects of this collaboration, a retrospective review was conducted for all patients who had suffered severe extremity trauma and received either a free or a pedicled flap for coverage of soft tissue defects 0–4 years before and after the established collaboration. The main hypotheses were that this collaboration would decrease the time to soft tissue coverage as well as the in-hospital stay.

## Material and methods

All consecutive patients who had suffered extremity trauma and who received either a free or a pedicled flap for coverage of soft tissue defects, between April 2004 and April 2012, were included. A comparison was made between the management of patients 4 years before the formalized collaboration (21 April 2004 – 20 April 2008) and 4 years after the formalized collaboration (21 April 2008 – 20 April 2012). Only patients with acute extremity trauma with exposed bone/open fracture or patients suffering from skin necrosis/infection after fracture surgery or chronic osteomyelitis following acute extremity trauma were selected for inclusion. Patients reconstructed after orthopedic tumor surgery or arthroplasty failures were not included. The included patients were identified by using the hospitals electronic operation planning system; Orbit (SYSteam Critical Care AB, Stockholm, Sweden). Patients were searched for using surgical procedure codes for different types of free and pedicled flaps. Time to flap coverage was counted from trauma/admission at Karolinska University Hospital, or in the secondary cases from occurrence of wounds, fistulation or exposed bone. In case of a failed flap with demand for a secondary flap, time to flap coverage was counted to the first flap.

Open fractures were classified according to Gustilo-Anderson [9]. Fractures were classified strictly after the status at admission. Fractures classified as GIIIA, which in secondary revisions developed into IIIB due to tissue necrosis or infection were not reclassified.

In order to investigate the impact of the collaboration on time to flap surgery after admission to the hospital, and the impact on the postoperative treatment and in-hospital stay, only patients with acute extremity trauma including open fractures or soft tissue defects with exposed bone were selected for a sub analysis (Tables 1 and 2). Patients with skin necrosis/infection after fracture surgery, chronic osteomyelitis, or patients referred to Karolinska University Hospital later than 14 days after the trauma were excluded from sub analysis.

**Table 1:** **Patients treated before start of multidisciplinary collaboration**

	**Sex**	**Age (y)**	**Case**	**Trauma**	**Gustilo**	**Flap**	**Time to flap (d)**	**Time to coverage (d)**
**Free flaps included**								
1	M	59	Distal tibia fracture	Excavator accident	GIIIB	Latissimus dorsi muscle	6	129
2	M	42	Proximal tibia fracture, fibula fracture, femoral fracture, humeral fracture	MC accident	GIIIB	Fibula osteocutaneous flap (Medial gastro-cnemius flap)	49 (0)	425 Failed Medial gastrocnemius flap
3	M	48	Distal femoral fracture	Bus accident	GIIIC	Latissimus dorsi muscle	7	Amputation 2 d after flap
**Free flaps excluded**								
4	M	54	Osteomyelitis calcaneal fracture 1 year earlier	MC accident	GIIIA	Gracilis muscle	360	7
5	M	21	Osteomyelitis proximal tibia after fracture 5 years earlier	Moped accident	GIIIB	Latissimus dorsi muscle	5 years	65
**Pedicled flap included**								
1	M	26	Multitrauma, calcaneal fractures with skin necrosis	Fall injury	GIIIA	Sural island flap	220	21
2	M	60	Diaphyseal tibia fracture	Fall injury	GIIIB	Fasciocutaneous rotation flap	26	52
**Pedicled flap excluded**								
3	M	64	Tibia condyle fracture and abundant soft tissue injury	Outboard motor accident	GIIIC	Lateral gastrocnemius muscle	26	72
4	F	30	Tibia pilon fracture, secondary skin necrosis after surgery	Fall injury	-	Sural island flap	38	4
5	F	54	Osteomyelitis, distal tibia fracture 2 years earlier, skin necrosis after secondary surgery	Fall injury	-	Fasciocutaneous transposition flap	21	Partial flap necrosis Free flap 2011 Pat 15 Table 2
6	F	27	Multitrauma, proximal tibia fracture, admitted 1 month after trauma	Car accident	GIIIB	Medial gastrocnemius muscle	51	57
7	M	26	Diaphyseal tibia fracture, admitted 1.5 months after trauma	Climbing accident	GIIIB	Soleus muscle	51	334

Patients treated before start of multidisciplinary collaboration with a free or pedicled flap for soft tissue reconstruction after lower extremity trauma. Patients excluded in the subgroup analysis of acute extremity trauma were patients recieving flaps due to skin necrosis/infection after fracture surgery, chronic osteomyelitis, or patients referred to Karolinska University Hospital later than 14 days after the trauma. Time to flap is counted from trauma/admission at Karolinska University Hospital, or in the secondary cases from occurrence of wounds, fistulation or exposed bone. Time to complete soft tissue coverage after flap surgery was determined as no remaining skin wounds at clinical evaluation at follow up.

**Table 2:** **Patients treated after start of multidisciplinary collaboration**

	**Sex**	**Age (y)**	**Case**	**Trauma**	**Gustilo**	**Flap**	**Time to flap (d)**	**Time to coverage (d)**
**Free flap included**								
1	M	31	Traumatic arm amputation	Work accident	GIIIC	Palmar free flap	0	75
2	F	33	Calcaneus fracture, ankle fracture, pelvic fracture	Run over by a lorry	GIIIB	Latissimus dorsi muscle	4	203
3	M	55	Diaphyseal tibia fracture with bone defect	Gunshot	GIIIC	Fibula osteo-cutaneous flap	4	100 Flap failure and amputation
4	M	42	Diaphyseal tibia fracture	Car accident	GIIIC	Anterolateral thigh flap	11	192
5	M	33	Multitrauma, diaphyseal tibia fracture	MC accident	GIIIC	Latissimus dorsi muscle	8	63
6	M	61	Distal tibia fracture	MC accident	GIIIC	Latissimus dorsi muscle	3	87
7	F	46	Distal tibia/fibula fracture	Riding accident	GII	Anterolateral thigh flap	7	31
8	F	31	Multitrauma, proximal tibia/fibula fracture	Bicycle accident	GIIIC	Latissimus dorsi muscle	16	148
9	M	20	Multitrauma, distal tibia fracture	Car accident	GIIIB	Latissimus dorsi muscle	6	23 Amputation 1 year after flap due to pain
10	M	27	Soft tissue defect tibia	MC accident	-	Gracilis muscle	7	104
11	M	34	Distal tibia fracture	MC accident	GIIIA	Gracilis muscle	29	35
12	M	66	Ankle fracture	Work accident	GIIIA	Anterolateral thigh flap	22	16
13	M	36	Distal tibia fracture	Car accident	GIIIA	Gracilis muscle	3	21
14	M	27	Multitrauma, tibia fracture with bone defect	MC accident	GIIIC	Fibula osteo- cutaneous flap	5	10
**Free flap excluded**								
15	F	60	Osteomyelitis, distal tibia fracture 6 years earlier	Fall injury	-	Anterolateral thigh flap	5.5 years	40 Pedicled flap 2006 Pat 5 Table 1
16	M	32	Infection after distal tibia fracture, referred 2 months after trauma	Car accident	GIIIB	Anterolateral thigh flap (Sural island flap)	120 (105)	42 Failed Sural island flap
17	F	53	Distal tibia fracture, secondary skin necrosis after surgery	Fall injury	-	Anterolateral thigh flap	456	29
18	M	76	Trimalleolar ankle fracture, referred 2.5 months after trauma	Fall injury	GIIIA	Gracilis muscle	105	128
19	M	44	Osteomyelitis, tibia fracture 15 months earlier	Moped accident	GIIIC	Latissimus dorsi muscle	432	24
20	F	63	Osteomyelitis, tibia pilon fracture 11 months earlier	Fall injury	-	Anterolateral thigh flap (Gracilis muscle)	295 (288)	134 Failed gracilis muscle
**Pedicled flap included**								
1	M	40	Proximal tibia fracture	MC accident	GIIIB	Propeller flap and lateral gastrocnemius muscle	3	457 Partial flap necrosis
2	M	70	Soft tissue injury to elbow, and humeral fracture	Lawn mower accident	GIIIB	Radial forearm flap	7	23
3	M	17	Soft tissue defect over knee	Moped accident	-	Medial gastrocnemius muscle	11	9
4	M	37	Proximal tibia fracture	Bus accident	GIIIA	Medial gastrocnemius muscle	51	28
5	M	31	Multitrauma, diaphyseal tibia fracture	Car accident	GIIIB	Fasciocutaneous rotation flap	4	16
6	M	65	Humeral fracture	Gunshot	GIIIB	Latissimus dorsi muscle	3	35
7	F	64	Proximal tibia fracture	Run over by a bus	GIIIA	Medial gastrocnemius muscle	16	65
8	F	15	Proximal tibia fracture	Moped accident	GIIIB	Medial gastrocnemius muscle	1	21
9	F	43	Multitrauma, soft tissue defect over patella	Train accident	-	Medial gastrocnemius muscle	34	10
10	M	55	Diaphyseal tibia fracture	Bicycle accident	GIIIB	Soleus muscle	4	4
11	M	36	Radial fractures	Gunshot	GIIIC	Fasciocutaneous transposition flap	0	15 Partial flap necrosis
**Pedicled flap excluded**								
12	F	59	Osteomyelitis, ankle fracture 5.5 years earlier	Fall injury	-	Extensor digitorum brevis muscle	5.5 years	112
13	M	55	Distal tibia fracture 7 years earlier, secondary skin necrosis after surgery	Fall injury	-	Extensor digitorum brevis muscle	7 years	49
14	M	47	Tibia pilon fracture, secondary skin necrosis after surgery	Fall injury	-	Sural island flap	28	143 Partial flap necrosis
15	M	55	Osteomyelitis, tibia condyle fracture 17 months earlier secondary skin necrosis after surgery	MC accident	-	Medial gastrocnemius muscle	156	8 Amputation 8 d after flap
16	F	71	Ulnar fracture, secondary skin necrosis after surgery	Fall injury	-	Fasciocutaneous rotation flap	63	18
17	F	86	Proximal tibia fracture, secondary skin necrosis after surgery	Fall injury	-	Medial gastrocnemius muscle	61	11
18	F	62	Distal tibia fracture 3 years earlier, wound infection after hardware removal	Fall injury	-	Soleus muscle	239	17 Partial flap necrosis
19	M	63	Multitrauma, patellar fracture, secondary skin necrosis after surgery	Car accident	-	Medial gastrochnemius muscle (Fasciocutaneous rotation flap)	(103) 51	190 Partial flap necrosis rotation flap
20	F	67	Distal tibia fracture, secondary skin necrosis after surgery	Fall injury	-	Propeller flap	135	Not healed by inclusion
21	M	76	Distal tibia fracture, secondary skin necrosis after plaster	Wheelchair accident	-	Sural island flap	42	Flap failure, Amputation 70 d. after flap

Patients treated after start of multidisciplinary collaboration with a free or pedicled flap for soft tissue reconstruction after lower extremity trauma. Patients excluded in the subgroup analysis of acute extremity trauma were patients recieving flaps due to skin necrosis/infection after fracture surgery, chronic osteomyelitis, or patients referred to Karolinska University Hospital later than 14 days after the trauma. Time to flap is counted from trauma/admission at Karolinska University Hospital, or in the secondary cases from occurrence of wounds, fistulation or exposed bone. Time to complete soft tissue coverage after flap surgery was determined as no remaining skin wounds at clinical evaluation at follow up.

Operations/revisions included all surgery performed in an operating room on the patient due to the trauma and included; primary wound debridement, primary fixation with external fixation device, secondary fixation in those cases where it did not concur with flap surgery, wound revisions/Topical negative pressure (TNP) therapy-change, reoperations due to flap failure, additional split skin grafting, extraction of external or internal fixation, bone grafting, conversion to circular external fixation (Taylor spatial frame™), corrections due to angulation/malrotation of fractures. TNP was only used as a dressing and not as a method to close the wound.

The total in-hospital stay was calculated by adding all admissions for operations/revisions before and after flap coverage.

The time to complete soft tissue coverage after flap surgery was determined as no remaining skin wounds at clinical evaluation at follow up and accepted as stated in the respective patient charts. Fracture healing was determined by radiological and clinical evaluation retrieved from patient charts.

The project was done in accordance with a protocol approved by the Ethical Committee at Karolinska Institutet, Stockholm, Sweden. (Project 2013/307-31/2).

The obtained results were statistically compared using a Mann–Whitney test in GraphPad Prism 5.0 (GraphPad Software Inc., CA). A p-value of ≤0.05 was considered statistically significant. Results were presented with median values.

## Results

In total, 52 patients (36 males, 16 females) treated with a free or pedicled flap were identified during the 8 year study period. One patient was included in both groups as she received one flap before and one after the start of the collaboration (53 cases). In total these patients received 57 flaps. 13 flaps were performed in the 4 years before the start of the collaboration, of which 5 were free flaps and 8 were pedicled flaps. 44 flaps were performed in the 4 years after the start of the collaboration, of which 21 were free flaps and 23 were pedicled flaps. Patients and choice of flaps is stated in Tables 1 and 2. All 5 free flaps performed before the collaboration survived. Total flap necrosis occurred in 2 out of 21 free flaps after the start of the collaboration. No partial necrosis was seen in free flaps. Total flap necrosis was seen in 1 pedicled flap before the start of the collaboration and 2 after the start of the collaboration. Partial flap necrosis was seen in 6 of the pedicled flaps (1 before and 5 after the start of the collaboration). The median age at the time of flap reconstruction before the start of the collaboration was 45 (21–64) years. The median age after start of the collaboration was 47 (15–86) years.

**Figure 1:**
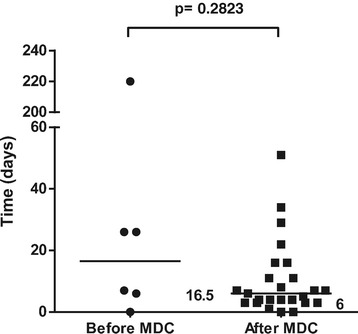
Time to flap surgery. Days from admission with acute extremity trauma at Karolinska University Hospital to flap surgery 4 years before and after start of multidisciplinary collaboration (MDC). Dots and squares represent individual patients. Six patients before the start of the MDC and 25 after the start of MDC were treated with either free or pedicled flaps due to acute extremity trauma with soft tissue defects.

**Figure 2:**
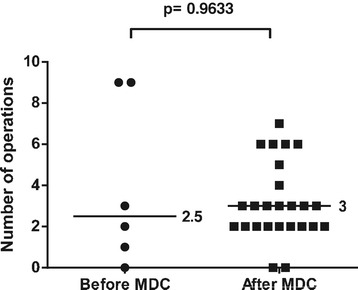
Number of revisions/operations prior to flap surgery. Number of revisions/operations prior to flap surgery in acute extremity trauma patients 4 years before and after start of multidisciplinary collaboration (MDC). Dots and squares represent individual patients. Six patients before the start of the MDC and 25 after the start of MDC were treated with either free or pedicled flaps due to acute extremity trauma with soft tissue defects.

**Figure 3:**
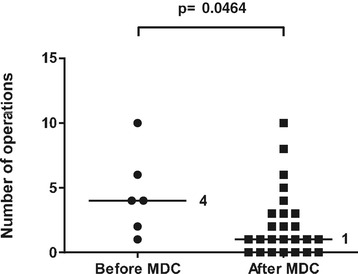
Number of revisions/operations after flap surgery. Number of revisions/operations after flap surgery in acute extremity trauma patients 4 years before and after start of multidisciplinary collaboration (MDC). Dots and squares represent individual patients. Six patients before the start of the MDC and 25 after the start of MDC were treated with either free or pedicled flaps due to acute extremity trauma with soft tissue defects.

**Figure 4:**
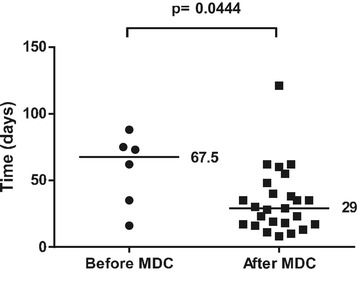
In-hospital stay. Days in hospital after admission with acute extremity trauma and treated with flaps in patients 4 years before and after start of multidisciplinary collaboration (MDC). Data includes all admissions, i.e. also secondary revisions. Dots and squares represent individual patients. Six patients before the start of MDC and 25 after the start of MDC were treated with either free or pedicled flaps due to acute extremity trauma with soft tissue defects.

In 6 out of 12 cases before the start of the collaboration and 25 out of 42 cases after the start of the collaboration, flap treatment was due to acute extremity trauma with soft tissue defects. The rest were skin necrosis/infection after fracture surgery, chronic osteomyelitis, or patients referred to Karolinska University Hospital later than 14 days after the trauma. These patients were excluded from the analysis of treatment of acute extremity trauma to achieve a more uniform cohort for comparison. Most fractures were graded as GIIIB, but there were also severe soft tissue injuries with exposed bone without fractures. The median time to flap coverage of acute extremity trauma was 6 (0–51) days after the start of the collaboration, compared to 16.5 (0–220) days before the start of the collaboration (p = 0.283) (Figure 1). The median number of revisions/operations prior to flap coverage of acute extremity trauma was 3 (0–7) after the start of the collaboration compared to 2.5 (0–9) before the start of the collaboration (p = 0.963) (Figure 2). The median number of revisions/operations following flap coverage of acute extremity trauma was 1 (1–10) after the start of the collaboration compared to 4 (0–10) before the start of the collaboration (p = 0.046) (Figure 3).The median time for complete soft tissue coverage after flap surgery of acute extremity trauma was 33 (9–203) days after the start of the collaboration compared to 62 (2–425) days before the start of the collaboration (p = 0.518). The median time in hospital after acute extremity trauma was reduced from 67.5 (16–88) days to 29 (8–121) days after the start of the collaboration (p = 0.044) (Figure 4). All included fractures healed. Time to fracture healing was 296 days before the start of the collaboration and 256 days after the start of the collaboration (p = 0.594). Only 5 out of 6 patients before and 16 out of 25 patients after the start of the collaboration were possible to follow to fracture healing. One patient was amputated after flap treatment before the start of the collaboration. After the start of the collaboration, four patients had soft tissue defects with exposed bone without fractures, one had a primary traumatic amputation, two were amputated prior to flap treatment and one patient was not healed at the time of inclusion in the study. One of the patients healed, but was amputated one year after flap coverage due to pain.

## Discussion

Severe extremity trauma with soft tissue defects is a difficult task for both the patient and health care provider. In order to optimize the outcome for the patients we have improved our care of the patients. There is a growing body of evidence that these complex extremity injuries are best handled by a multidisciplinary team of experienced plastic and orthopedic surgeons [8,10]. The present study clearly demonstrates an increase of flap reconstruction in acute trauma patients and patients with secondary wounds following acute extremity trauma after the start of a multidisciplinary collaboration. This is hardly explained by natural variation, as the observation time is 8 years, and it seems unlikely that variation could explain a 3.5-fold increase as in our series. In 2007 all high energy trauma in Stockholm County was regionalized to Karolinska University Hospital. This has resulted in a 0.75-fold increase of trauma patients. Patients with severe extremity trauma is now primary taken to Karolinska University Hospital. Prior to 2007 the reference policy was less strict and hence not all cases were treated at Karolinska. The severity of orthopedic trauma has not changed during the years. Occasionally patients with open fractures are still treated in other hospitals in Stockholm, but they are usually referred to Karolinska after external fixation and primary revision. We have also seen an increase in both acute and secondary cases referred from other hospitals in the region as the collaboration has become known. The regionalization only cannot explain a 3.5-fold increase of flaps. We believe that the main reason is an increased awareness among our orthopedic surgeons to refer extremity trauma patients for plastic surgical consultation. The collaboration has also resulted in a more rapid communication, and quicker decisions.

The major limitation in this report is the small sample size. Since the patient group treated before start of the collaboration is small, the sub analysis of acute extremity trauma patients cannot provide accurate statistical comparison, and the reader should interpret the significance between groups with care. One of the acute trauma patients included before start of the MDC was not covered before 220 days after trauma. This patient had bilateral calcaneal fractures after a fall injury, classified as a Gustilo IIIA due to crush wounds on the heels. During the following days he developed skin necrosis on the right heal. Due to a more conservative attitude towards small wounds in extremity trauma before the start of MDC, there was an attempt of secondary healing. Today, this patient would probably have received a free flap in the near time period of the trauma. To reduce the effects of this “outlier” statistically we have chosen to use a nonparametric method, and present median values. This patient was not an outlier in comparisons other than time to flap.

The median time to flap coverage after the start of the collaboration was 6 days, which still is not satisfactory. Institutional factors such as available OR time and support staff may make immediate reconstruction of extremity injuries impossible. There can also be other concomitant injury which first has to be dealt with, and the extent of soft tissue injury is sometimes difficult to determine early in the process. Whereas Godina [1] clearly states the superiority of reconstruction within 3 days, other authors have shown excellent results with late coverage [11-13]. There was no difference in outcome or flap failure in our series when comparing subgroups of patients; flap coverage < 3 days vs. > 3 days, or flap coverage < 6 days vs. > 6 days (data not shown). It is not possible to draw overreaching conclusions from this due to the relative small groups in comparison to the work by Godina [1]. One key to earlier flap coverage is to perform fewer revisions. After the start of the collaboration we still had a median number of 3 operations/revisions before flap coverage. Only one radical debridement performed by the most experienced surgeons before flap coverage has been set up as a future goal for the team.

The introduction of a collaboration had a significant impact on the in hospital stay. The median time in hospital after acute extremity trauma with soft tissue defects decreased from 68.5 days to 29 days. In the 25 patients after the start of the collaboration, this would correspond to 988 hospital days, and a total cost reduction of 1.170 000 USD. In times where cost-analysis is crucial in public health care, this reduction which corresponds to 70% of one hospital bed/year is obviously beneficial. The reduction of hospital stay may be explained by the decrease in revisions/operations after flap coverage, which may be related to a shorter time to coverage.

The incidence of open tibia fractures in the Swedish population is 2.3/10^5^/year [6], which corresponds to about 220 patients per year, of which 27% [5] have a Gustilo Type III fracture. Because of the low and declining incidence, centralization of these difficult fractures has been suggested [8,14]. We believe that these fractures should be dealt with in a center with a close collaboration between orthopedic and plastic surgeons. The experience after the Haiti earthquake with an orthoplastic limb salvage team are in concordance, showing favorable amputation rate and a more efficient planning of surgical workload [10].

## Conclusion

To conclude, the formalized collaboration has led to an increased number of flap coverage in extremity trauma patients. The acute cases have a shorter in-hospital stay, and fewer postoperative revisions after the start of the collaboration. However, if this has resulted in a decreased number of amputations and improved long-term outcome needs to be further analyzed in a larger cohort. Our collaboration has also led to awareness among our orthopedic colleagues to establish an early contact with a plastic surgeon when a patient has an open fracture with soft tissue damage. The goal is that the plastic surgeon should be contacted from the emergency room in order to participate at the first operative revision to be able to plan for further surgery. Routines are also created for coordination of operative resources, and postoperative flap surveillance. Finally, this formal collaboration gives us better possibilities for quality control and evaluation. Our experience leads us to recommend other centers to establish a similar set-up for early multidisciplinary treatment of this patient group.

## Consent

Written informed consent was obtained from the patients for the publication of this report.
